# Ubisol-Q_10_, a Nanomicellar and Water-Dispersible Formulation of Coenzyme-Q_10_ as a Potential Treatment for Alzheimer’s and Parkinson’s Disease

**DOI:** 10.3390/antiox10050764

**Published:** 2021-05-11

**Authors:** Darcy Wear, Caleb Vegh, Jagdeep K. Sandhu, Marianna Sikorska, Jerome Cohen, Siyaram Pandey

**Affiliations:** 1 Department of Chemistry and Biochemistry, University of Windsor, 401 Sunset Avenue, Windsor, ON N9B 3P4, Canada; wear@uwindsor.ca (D.W.); veghc@uwindsor.ca (C.V.); 2 Human Health Therapeutics Centre (HHT), National Research Council Canada, 1200 Montreal Road, Ottawa, ON K1A 0R6, Canada; 3 Department of Biochemistry, Microbiology and Immunology, University of Ottawa, 451 Smyth Road, Ottawa, ON K1H 8M5, Canada; 4 Researcher Emeritus, Human Health Therapeutics Centre, National Research Council Canada, 1200 Montreal Road, Ottawa, ON K1A 0R6, Canada; marianna.sikorskawalker@gmail.com; 5 Department of Psychology, University of Windsor, 401 Sunset Avenue, Windsor, ON N9B 3P4, Canada; jcohen@uwindsor.ca

**Keywords:** progressive neurodegeneration, astroglia activation, oxidative stress, antioxidant, vitamin E, mitochondria, senescence, inflammation, autophagy, apoptosis

## Abstract

The world continues a desperate search for therapies that could bring hope and relief to millions suffering from progressive neurodegenerative diseases such as Alzheimer’s (AD) and Parkinson’s (PD). With oxidative stress thought to be a core stressor, interests have long been focused on applying redox therapies including coenzyme-Q_10_. Therapeutic use has failed to show efficacy in human clinical trials due to poor bioavailability of this lipophilic compound. A nanomicellar, water-dispersible formulation of coenzyme-Q_10_, Ubisol-Q_10_, has been developed by combining coenzyme-Q_10_ with an amphiphilic, self-emulsifying molecule of polyoxyethanyl α-tocopheryl sebacate (derivatized vitamin E). This discovery made possible, for the first time, a proper assessment of the true therapeutic value of coenzyme-Q_10_. Micromolar concentrations of Ubisol-Q_10_ show unprecedented neuroprotection against neurotoxin exposure in in vitro and in vivo models of neurodegeneration and was extremely effective when delivered either prior to, at the time of, and most significantly, post-neurotoxin exposure. These findings indicate a possible way forward for clinical development due to effective doses well within Federal Drug Administration guidelines. Ubisol-Q_10_ is a potent mobilizer of astroglia, antioxidant, senescence preventer, autophagy activator, anti-inflammatory, and mitochondrial stabilizer. Here we summarize the work with oil-soluble coenzyme-Q_10_, its limitations, and focus mainly on efficacy of water-soluble coenzyme-Q_10_ in neurodegeneration.

## 1. Introduction

Various mechanisms have been implicated in the progression of neurodegenerative processes. These include excessive oxidative stress, mitochondrial dysfunction, autophagy deficiencies, protein aggregation and misfolding, inflammation, excitotoxicity, cell death pathways, and loss of trophic support. However, none of these mechanisms have been proven to be a primary cause of neurodegenerative disorders [1,2,3]. This is likely because these pathogenic pathways are engaged at different stages of disease progression and are often a secondary manifestation of the disease process. Accordingly, no single drug and/or therapeutic approach to mitigate the neurodegeneration derived from various preclinical studies has been successful so far, but the needs are urgent and critically important.

It has long been recognized that CoQ_10_, a natural lipid soluble antioxidant and enzyme cofactor, possesses many molecular features that could play a role in neuroprotection. CoQ_10_ is an essential constituent of cell membranes where it controls cellular redox states as a critical cofactor of cellular oxidoreductases. Its function is essential for cellular homeostasis and viability. It can act as either a two electron or one electron carrier during the transition from fully oxidized (ubiquinone) to fully reduced (ubiquinol) forms. It is critical not only for mitochondrial electron transport chains and cellular energy production, but also for the function of many cellular oxidoreductases [4]. It is also a powerful antioxidant and free radical scavenger [5,6]. Although it is produced by cells, its content is low, decreasing with age or disease. The efforts to increase the cellular content through supplementation are mostly unsuccessful due to its lipophilic nature. Within the scientific communities, very significant efforts have been made to solubilize and increase the absorption/bioavailability of supplemented CoQ_10_. Multiple methods have been developed and tested and these include various approaches to emulsify and/or disperse CoQ_10_ and more recently methods to achieve water solubility which are extensively reviewed [7,8,9,10]. Most of these formulations have been shown to have an increased cellular absorption/bioavailability; however, none is suitable for parenteral delivery due to drawbacks of drug emulsions such as rate of dispensation, degree of emulsification, particle size, or drug precipitation from the formulation upon dispersion.

## 2. Standard Oil-Soluble Formulation of Coenzyme-Q_10_ in Neurodegenerative Diseases

A significant amount of work has been done with oil-soluble coenzyme-Q_10_ and many excellent reviews have been written on the subject focusing on general human health [11,12] and neurodegenerative diseases [13,14,15,16] such as Parkinson’s disease [17,18,19]. Oil-soluble coenzyme-Q_10_ has been studied as a potential therapeutic for many neurodegenerative diseases including Parkinson’s disease, Alzheimer’s disease, Huntington’s disease, amyotrophic lateral sclerosis, among others [20]. The next section will briefly discuss previous results testing oil-soluble CoQ_10_ on these various neurodegenerative diseases.

### 2.1. Parkinson’s Disease

Mitochondrial dysfunction and excessive reactive oxygen species (ROS) have been shown to be major factors in the development of Parkinson’s disease. Initially, Beal et al. used a 1-methyl-4-phenyl-1,2,3,6-tetrahydropyridine (MPTP)-induced mouse model of PD and demonstrated the mitigation of MPTP-induced loss of striatal dopamine and dopaminergic axons in mice treated with 200 mg/kg/day CoQ_10_ in their diet (Table 1) [21]. Further investigation by this group using a formulation of CoQ_10_ with surfactant (Tishcon CoQ_10_) demonstrated significant neuroprotection at doses of 1600 mg/kg/day in MPTP mice [22]. Administration of normal oil-soluble CoQ_10_ at 1600 mg/kg/day through diet significantly reduced the loss of dopaminergic neurons in the substantia nigra pars compacta (SNpc) while preventing the formation of α-synuclein aggregates in dopaminergic neurons [22]. Combining CoQ_10_ (1% of diet) with creatine (2% of diet), shown to protect against excitotoxicity and β-amyloid toxicity in vitro [23], researchers demonstrated additive neuroprotective effects against MPTP neurotoxicity in mice [24]. This combination was able to reduce lipid peroxidation, the accumulation of α-synuclein in SNpc neurons, and dopaminergic neuron loss in vivo. Examining various antioxidants on a Drosophila model of Parkinson’s disease, Faust et al. found no neuroprotection following CoQ_10_ supplementation at very high doses of 100 mg/mL in their drinking water (Table 1) [25]. This group obtained approval for a clinical trial using the Tishcon CoQ_10_ formulation; however, the maximum dose given to the patient as approved by the FDA was far lower than that reported in the in vivo work. Therefore, it is not surprising that this formulation did not show any significant effects in patients [26]. A formulation with bioavailability and efficacy at FDA-approved doses should be evaluated and advanced to a clinical study.

In an attempt to improve the bioavailability of CoQ_10_, Park et al. used direct intrastriatal delivery of oil-soluble CoQ_10_ [27]. Using 6-hydroxydopamine (6-OHDA)-induced Parkinson’s rats, they compared the progression of cell death in the substantia nigra (SN) region of the brain between the rats receiving intrastriatal CoQ_10_ and rats receiving oral administration of CoQ_10_ at significantly higher doses. Results indicated increased tyrosine hydroxylase expression in the striatum and SN as well as a reduction in TNF-α, a pro-inflammatory cytokine, following intrastriatal delivery of CoQ_10_ [27]. This demonstrates the targeting and amelioration of two critical Parkinsonian pathologies, those being the loss of tyrosine hydroxylase positive neurons in the SN and neuroinflammation. These results indicated considerable neuroprotection at doses significantly lower than what is seen with oral delivery of oil-soluble CoQ_10_ (Table 1). Furthermore, this study helps demonstrate the importance of increasing the bioavailability of CoQ_10_ as it can result in significantly improved neuroprotection. Although it gave good results, intrastriatal injections are not a conceivable way to treat patients.

### 2.2. Alzheimer’s Disease

Oxidative stress and mitochondrial dysfunction are also critical causative features of Alzheimer’s disease development [28,29]. Researchers have investigated the therapeutic properties of oil-soluble coenzyme-Q_10_ both in vitro and in vivo. MC65 cells are a human neuroblastoma cell line expressing residues of the amyloid precursor protein (APP), commonly cleaved by secretases to form amyloid-β plaques in AD patients [30,31,32]. Woodworth et al. used hydrophobic CoQ_10_ solubilized in ethanol in MC65 cells and demonstrated the neuroprotective effects with a dose of 6.25 µM (around 5 mg/mL, a relatively high dose) against neurotoxicity and oxidative stress (Table 1) [30]. Another research group examined the effects of 10 µM CoQ_10_ (dissolved in acetone) against Aβ- and zinc-induced mitochondrial dysfunction in M17 human neuroblastoma cells (Table 1). They demonstrated CoQ_10_′s ability to restore zinc-mediated cellular dysfunction and provide neuroprotection against the associated mitochondrial dysfunction [33]. It was also shown that CoQ_10_ can stabilize the membrane of neuronal cells following Aβ-induced alterations in membrane potential [33]. Furthermore, in vitro application of CoQ_10_ has reported its ability to prevent the accumulation of the characteristic Aβ aggregates and thus inhibit its toxicity [34].

Interestingly, in vivo studies with oral supplementation of 10 g/kg diet of CoQ_10_ have shown antioxidative effects in the brains of wild-type mice; however, the levels of mitochondrial CoQ_10_ were not increased in their brains casting doubt about the blood-brain-barrier permeability and brain bioavailability (Table 1) [30]. Furthermore, using a Tg19959 transgenic mouse model of AD, Dumont et al. showed that oral CoQ_10_ supplementation reversed AD pathologies through the reduction of oxidative stress and Aβ42 levels, while improving cognitive performance (Table 1) [35]. Overall, researchers have shown the neuroprotective efficacy of CoQ_10_ and its ability to target multiple pathologies associated with Alzheimer’s disease. Unfortunately, the daily CoQ_10_ intake required to show this efficacy is very high and cannot be translated to human patients. If an improved formulation were created that can increase bioavailability particularly in the brain, it could permit the use of more biologically relevant doses experimentally and clinically to treat AD.

### 2.3. Huntington’s Disease

Huntington’s disease (HD) occurs due to neurodegeneration in the striatum [36] with mitochondrial dysfunction and increased oxidative stress playing crucial roles in its pathogenesis [37,38]. Furthermore, the misfolding and aggregation of the Huntington (HTT) protein indicates that a supplement which can improve mitochondrial function and eliminate oxidative stress while activating autophagy may be beneficial in the treatment of HD [38]. Specifically, Matthews et al. found that oral supplementation of 200 mg/kg/day CoQ_10_ was effective in reducing 3-NP associated neurotoxicity in rat models of HD (Table 1) [39]. In transgenic mouse models of HD, 400 mg/kg/day CoQ_10_ supplementation was shown to significantly increase survival while influencing disease progression as well (Table 1) [40]. CoQ_10_ intake led to improved motor performance, mitigated the formation of striatal neuron intranuclear inclusions, delayed reductions in body weight, and slowed neuronal and gross brain atrophy in the striatum [40]. Combining a diet of 0.2% CoQ_10_ and minocycline, Stack et al. demonstrated improved survival, behavioral performance, and improved biochemical pathologies including brain atrophy, neuronal atrophy, and HTT aggregation compared to either treatment alone in R6/2 transgenic mice (Table 1) [41]. Examining weight loss, a common feature of HD patients and R6/2 transgenic mice [42], CoQ_10_ alone demonstrated drastic improvements, even compared to the combined treatment [41]. It could be that HD-associated weight loss occurs as a result of energy metabolism defects which are being ameliorated by CoQ_10_ [41]. Other combinatorial studies examined high oral doses of CoQ_10_ in combination with creatine on a 3-NP rat model of HD and R6/2 transgenic mice (Table 1) [24]. Additive neuroprotective effects were observed whereby striatal lesion volume decreased, glutathione homeostasis was maintained, and oxidative damage was attenuated following 3-NP administration. The expected increase in motor performance and survival were demonstrated in the R6/2 transgenic mice [24]. Unfortunately, the dosing used in this experiment is extremely high and unrealistic for humans corresponding to approximately 1 kg of CoQ_10_ per day for a 62 kg individual (the average weight worldwide). As a model of the more common middle-age onset form of HD, Hickey et al. used CAG140 knock-in mice to better demonstrate the progressive nature of the disorder. Feeding the mice with 0.2% or 0.6% CoQ_10_ in their diet (Table 1), they observed improvements in behavior deficits without blocking huntingtin protein aggregation in the striatum [43]. If another formulation were able to more readily cross the blood-brain-barrier increasing bioavailability in the brain, this could aid in the clearance of these protein aggregates. Interestingly, they found more benefits using the lower dose of 0.2% compared to 0.6%. Furthermore, 0.2% CoQ_10_ led to deleterious effects in WT mice whereby open field rearing and activity and rotarod performance were reduced; however, the absence of these deleterious effects in the mutant mice along with the outstanding safety profile of CoQ_10_ in humans may render this result insignificant [43]. Overall, the antioxidant and mitochondrial stabilizing properties of CoQ_10_ make it an excellent candidate to treat the progression of Huntington’s disease if clinically relevant doses can be achieved.

### 2.4. Amyotrophic Lateral Sclerosis

Amyotrophic Lateral Sclerosis (ALS) is another neurodegenerative disease characterized by progressive muscle weakness and atrophy, as well as the loss of motor neurons [44]. Mutations in superoxide dismutase (SOD1) commonly associated with ALS implicate the role of oxidative stress in its pathogenesis [45]. As a result, coenzyme-Q_10_ with its antioxidant and neuroprotective abilities may serve as a potential therapeutic to halt the progression of ALS. Using transgenic rat models of ALS, oral supplementation of 200 mg/kg/day of CoQ_10_ (Table 1) led to a subsequent increase in coenzyme-Q_9_ and coenzyme-Q_10_ in the cerebral tissues and increases in mitochondrial CoQ_10_ concentrations [39]. In a transgenic mouse model of familial ALS containing a point mutation in the SOD1 gene, known to lead to motor neuron degeneration in ALS patients [46], mitochondrial dysfunction of motor neurons has been observed [47]. However, oral supplementation of CoQ_10_ resulted in antioxidant effects and preserved mitochondrial function in SOD1-mutated transgenic mice resulting in an overall increase in their lifespan [39]. Unfortunately, similar studies on SOD1^G93A^ mice demonstrated no effect on disease progression following oral supplementation of 200 mg/kg/day CoQ_10_ (Table 1) [44]. As endogenous CoQ_10_ levels are significantly increased in these ALS mice in an attempt toward a protective antioxidant state, it could be that the supplementation of exogenous CoQ_10_ has little to no additional effects on CoQ_10_ levels in the brain. However, if the bioavailability of CoQ_10_ is improved, it could allow for important neuroprotection in the Central Nervous System (CNS) of ALS models and patients providing a potential therapeutic treatment.

### 2.5. Other Neurodegenerative Diseases

Various other neurodegenerative diseases present similar hallmarks to the aforementioned conditions including oxidative stress and mitochondrial dysfunction, ultimately resulting in neuronal death. Frontotemporal dementia is characterized by progressive atrophy of the frontal and temporal lobes as well as neurofibrillary tangles (NFT) [48,49]. Using P301S mice containing a mutation in the tau protein, resulting in NFT generation and tau hyperphosphorylation, Elipenahli et al. demonstrated improved survival and behavior without significantly affecting tau hyperphosphorylation levels following supplementation of CoQ_10_ comprising 0.5% of the mouse diet (Table 1) [50].

Machado-Joseph Disease (MJD) or spinocerebellar ataxia type 3 (SCA3) is a neurodegenerative disease caused by Cytosine-Adenine-Guanine (CAG) triplet repeat expansions which result in an expanded polyglutamine tract in the ataxin-3 (ATX3) protein [51]. This results in protein misfolding, dysfunction and aggregation resulting in neuronal cell death [51,52]. Using PC12 cells transfected with expanded ATX3 as a model of MJD, treatment with 10µM CoQ_10_ was shown to improve cell viability, reduce the percentage of apoptotic cells, and prevent ATX3 protein aggregation ultimately ameliorating MJD-like pathologies (Table 1).

Multiple System Atrophy (MSA) is another neurodegenerative disease which is characterized by autonomic failure with combinations of parkinsonism, cerebellar ataxia, and pyramidal dysfunction [53]. Patients with MSA often display oligodendrocytes containing α-synuclein aggregates and neurons containing apoptotic proteins [54,55]. Furthermore, decreased levels of CoQ_10_ have been observed in the cerebellum of MSA patients [56]. As a result, Nakamoto et al. obtained induced pluripotent stem cells (iPSCs) from MSA patients and differentiated them into neurons. These cells were then supplemented with 25µM CoQ_10_ (Table 1) and showed improved mitochondrial oxidative metabolism and reduced amounts of apoptosis [53].

## 3. Water-Soluble Coenzyme-Q_10_

Various water-soluble formulations of coenzyme-Q_10_ have been developed in the hopes of increasing bioavailability for the treatment of human diseases. Prosek et al. water solubilized coeznyme-Q_10_ through the encapsulation of CoQ_10_ in the lipophilic cavity of β-cyclodextrin and demonstrated increased bioavailability in beagle dogs [64]. Micellar aqueous CoQ_10_ was generated by combining oil-soluble coenzyme-Q_10_ with polyethylene glycol monostearate (stPEG) and demonstrated higher blood circulation in mice compared to oil-soluble formulations [65]. Supplementation of another water-soluble form of coenzyme-Q_10_ named Qter in H9c2 cardiomyocytes or T67 astrocytoma cells resulted in elevated mitochondrial and cellular ubiquinone content compared to oil-soluble CoQ_10_ supplementation while further enhancing mitochondrial activity and reducing oxidative stress [66]. Using a water-soluble formulation on murine hippocampal HT22 cells, rotenone induced toxicity was severely depleted whereby water-soluble coenzyme-Q_10_ reduced oxidative stress, stabilized mitochondria, and prevented apoptosis-inducing factor (AIF) translocation and subsequent cell death [67]. Cui et al. developed their own water-soluble formulation from a glycyrrhizic-carnitine mixed layer CoQ_10_ micelle based on acyltransferases and tested it against oil-soluble coenzyme-Q_10_ in a rat model of chronic tacrolimus nephropathy. Water-soluble CoQ_10_ showed elevated blood stream levels compared to the oil-soluble formulation while reducing oxidative stress and apoptosis levels [68]. Water-soluble formulations have also been tested clinically and shown promise in trials for fibromyalgia where it reduced symptoms including pain and fatigue [69] and in patients suffering from presbycusis where significant improvements were observed compared to groups receiving vitamin E or a placebo [70]. Although these formulations of water-soluble coenzyme-Q_10_ have been tested on various in vitro and in vivo models as well as in clinical trials in certain cases, most works done on neurodegenerative diseases have used the Ubisol-Q_10_ formulation of water-soluble CoQ_10_ described in the next section.

## 4. Ubisol-Q_10_ Formulation and Its Properties

Micellization is a well-known process in the art of drug delivery and is applied to many lipid soluble compounds, i.e., vitamins (vitamin E, D3, K) and many novel drugs [71,72], as they become more readily absorbed by the body. The water-soluble products have enhanced bioavailability, stability, efficacy, and safety [7,8,9,10,73]. Recently Wang and Hekimi (2020) reported a simple two component nanomicellar formation of CoQ_10_ with an antifungal drug caspofungin. The researchers discovered that the synthetic drug caspofungin can form stable micelles and delivers better bioavailability of CoQ_10_ [74]. In their formulations, the antifungal pharmaceutical drug is combined with CoQ_10_ at a ratio of 0.225 µM:10 or 20 µM and forms wide size distribution micelles with an average size of ~40 nm. Our formulation is simple, it delivers simultaneously two bioactive compounds, vitamin E and CoQ_10_, of well-known biological activities, at a molar ratio of 2 moles vitamin E per 1 mol CoQ_10_.

**Figure 1:**
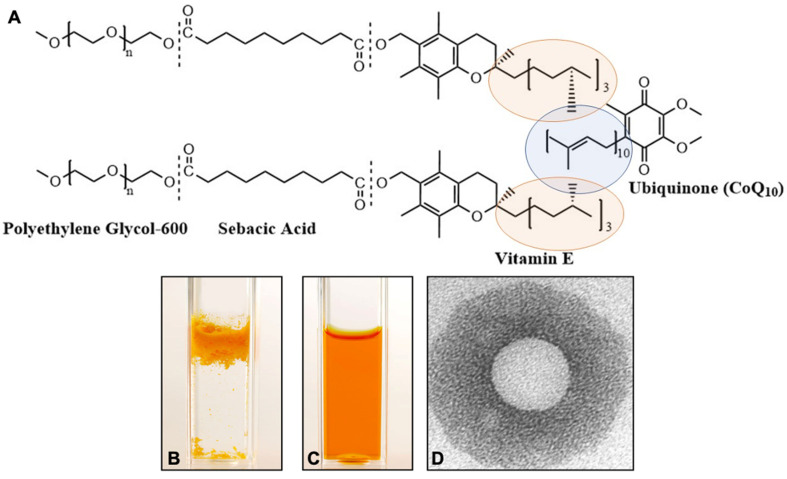
Ubisol-Q_10_ formulation. (**A**) chemical composition of the formulation consisting of 2 molecules of PTS per 1 molecule of CoQ_10_; (**B**) a direct suspension of 100 mg CoQ_10_ in water; (**C**) the same quantities of CoQ_10_ formulated with PTS as Ubisol-Q_10_ (clear and transparent solution of Ubisol-Q_10_ stable for years); (**D**) Transmission electron micrograph of a single Ubisol-Q_10_ nanomicelle measuring on average 22 ± 7 nm in diameter.

The National Research Council of Canada researchers have created Ubisol-Q_10_, a water-dispersible CoQ_10_ formulation allowing an easy and quantitative delivery of CoQ_10_. This discovery made possible, for the first time, a proper assessment of its true therapeutic value and potential mechanisms of action. In the pursuit to improve solubility and uptake of CoQ_10_, the researchers have taken lessons from mechanisms of bodily absorption of lipids and physiological roles of Coenzyme Q_10_. In the human body, the absorption and uptake of CoQ_10_ involves secretion into the small intestine with pancreatic enzymes and bile, which facilitates emulsification and micelle formation, typically required for absorption of lipophilic substances. Furthermore, in cellular membranes, CoQ_10_ interacts with vitamin E (α-tocopherol) and participates in its regeneration to facilitate free radical scavenging efficacy [73]. It has been discovered that α-tocopherol can be transformed into an amphiphilic self-emulsifying molecule of PTS (polyoxyethanyl α-tocopheryl sebacate). PTS can be synthesized by joining polyoxyethanyl-600 and α-tocopherol to decanedioic (sebacic) acid. The PTS molecule was created using well-known and generally regarded as safe (GRAS) components and it was granted GRAS status by the USA FDA. To achieve a water-dispersion, researchers have subjected CoQ_10_ to the micellization procedure and created Ubisol-Q_10_, a water-dispersible nanomicellar formulation of CoQ_10_, which actually consists of 2 components: CoQ_10_ and polyoxyethanyl-α-tocopheryl sebacate (PTS), combined at a ratio 1:2 mol/mol (Figure 1). The PTS molecule is an amphiphile, possessing both hydrophilic (PEG-600) and lipophilic (α-tocopherol) properties, separated by an aliphatic spacer sebacic (decanedioic) acid, and has self-emulsifying properties [75,76,77,78]. PTS is a waxy semisolid substance and similarly to coenzyme-Q_10_, has a low melting temperature (close to 50 °C); hence both substances could be co-melted to combine and form stable uniform nanomicelles when dispersed in water. When combined with water, the formulation can be heated to the boiling point of water and hence sterilized, and it is ready for parenteral delivery. In these studies, it has been tested mainly as a drinking tonic, and this form of application was sufficient to block the ongoing neurodegeneration in animal models, but it also could be easily injected (for example in stroke) [79]. A transmission electron microscopy analysis revealed that a single PTS-CoQ_10_ micelle measures 22 ± 7 nm in diameter. This Ubisol-Q_10_ formulation remained as a stable clear solution for ≥2 years, even at room temperature, and rendered CoQ_10_ fully dispersed in water and easily absorbed by the body as demonstrated by the elevated plasma and brain contents of CoQ_10_ within 1 h of Ubisol-Q_10_ ingestion. Thus, a new potent formulation has been created named Ubisol-Q_10_, a water-dispersible coenzyme-Q_10_, allowing easy and quantitative delivery of CoQ_10_ [60,61,62,63,79,80,81].

Upon systematic delivery, the PTS is converted back to α-tocopherol, CoQ_10_ is released and both compounds, vitamin E and ubiquinone are readily absorbed and find their way into the brain where they evoke powerful neuroprotection. They do not accumulate in tissues, but they provide neuroprotection for as long as they are continuously provided [60,61,62,63,79,81]. This seems to be a perfect solution for the treatment of ongoing neurodegeneration and will help millions of patients for whom there is little or no hope now.

In this review, we summarized up to date research results pertaining to the mechanism of action and efficacy of neuroprotection by Ubisol-Q_10_ in both in vitro and in vivo studies.

## 5. Ubisol-Q_10_ Protects Differentiated Neuronal Cells from Oxidative Stress and Excitotoxicity

CoQ_10_ (oil-soluble formulation) could not be properly tested in cell culture models, as it floats on top of aqueous media (Figure 1). The application of lipofection for internalization of CoQ_10_ in differentiated neuronal cell culture leads to a degeneration of neuronal extensions and neuronal cell death. CoQ_10_ is partially soluble in ethanol, but the addition of ethanol to cell media, particularly in larger volumes to ensure delivery of correct concentration of CoQ_10_, can denature the cellular components leading to solvent induced cell death. The bioavailability and water solubility of Ubisol-Q_10_ (Figure 1) allowed us to perform successful experimentation on neuronal cell cultures and examine the effects of direct oxidative stress (H_2_O_2_ treatment), hypoglycemia, hypoxia and glutamate excitotoxicity, as well as paraquat (PQ) neurotoxicity [82,83,84]. The results show that Ubisol-Q_10_ can significantly reduce the oxidative stress burden, contribute to the maintenance of the cellular Adenosine Triphosphate (ATP) pool, stabilize the mitochondrial membrane potential, and ultimately protect differentiated neurons. Typically, the exposure of differentiated human teratocarcinoma (NT2) and neuroblastoma (SH-SY5Y) cells to hydrogen peroxide leads to excessive cellular ROS generation and apoptotic cell death. However, pre-treatment of the neuronal cells with Ubisol-Q_10_ inhibited ROS production, stabilized the mitochondrial membrane, and prevented apoptosis [82].

Researchers have also investigated the cytotoxic effects of glutamate on cell cultures consisting of NT2/N neurons and NT2/A astrocytes derived from human NT2/D1 cells. In these experiments the cultures were exposed to 0.5 mM glutamate and 6 h of hypoxia. Neuronal cell death occurred during subsequent periods of reoxygenation [83]. Significantly, when these cells were pretreated for 72 h with Ubisol-Q_10_ before glutamate/hypoxia exposure, there was a decrease in ROS, increase in ATP production, and neuronal cells were protected, suggesting the incorporation of CoQ_10_ into cellular sub-compartments created an environment that could neutralize the effects of these neurotoxic treatments [83].

Furthermore, McCarthy et al. demonstrated that paraquat, an herbicide and mitochondrial toxin, caused increases in oxidative stress, and induced apoptosis in differentiated neuroblastoma cells [84]. The authors observed that treatment with paraquat led to increased ROS generation, depolarization of the inner mitochondrial membrane, and cell death. Importantly, pre-treatment with Ubisol-Q_10_ provided near complete protection against paraquat toxicity through inhibition of ROS generation and preventing the collapse of the mitochondrial membrane potential in these differentiated neuroblastoma cells [84]. These findings are very important as exposure to paraquat has been shown to be corelated with an increased risk of Parkinson’s disease.

## 6. Ubisol-Q_10_ Inhibits Oxidative Stress and Premature Senescence while Inducing Autophagy in Human Fibroblasts Obtained from AD Patients

It has been well established that mutations in the gene coding for presenilin-1 (PS-1) are a risk factor that predispose for development of Alzheimer’s disease. Although human neuron models of PD exist using various toxic insults, obtaining neurons from AD patients with the PS-1 mutation is not possible. Although not neurons, skin cells/fibroblast are very easy to obtain from AD patients who have the PS-1 mutation. In principle, these fibroblasts have the same PS-1 mutation as a neuron would from the same patient and at the biochemical level, the PS-1 mutation should have the same effect on fibroblasts similar to neurons. PS-1 mutated fibroblasts (PSAF) obtained from Alzheimer’s disease patients serve as a useful model for analyzing the induction of senescence and autophagy, as well as studying the relationship between them which is not well understood. A strong link between PS-1 and functional autophagy has been demonstrated with autophagy deficiencies observed in AD-related PS-1 mutations [85]. The PS-1 mutation results in constant endogenous ROS generation and leads to the inhibition of autophagy in these cells. Cellular stressors in PSAF such as endogenous ROS result in the overexpression of cell cycle arrest proteins p21, p53, and p16Ink4A [57]. Furthermore, retinoblastoma (Rb), a protein involved in cell cycle regulation shows higher expression levels in PSAF compared to normal human fibroblasts (NHF). These characteristics lead to the induction of stress-induced premature senescence (SIPS) in PSAF. Interestingly, growing PSAF in the presence of Ubisol-Q_10_ led to the potent inhibition of oxidative stress while lowering cell cycle arrest protein expression levels and preventing the onset of SIPS. Differential gene expression profiling demonstrated the down-regulation of autophagy-related genes in PSAF compared to NHF [59]. However, Ubisol-Q_10_ treated PSAF demonstrated gene expression profiles with an up-regulation of autophagy-related genes in a manner comparable to NHF. In particular, autophagy-related genes beclin-1 and JNK1, which are down-regulated in PSAF compared to NHF, were shown to be drastically up-regulated in PSAF following Ubisol-Q_10_ treatment at both the gene and protein level. Autophagy is known to be an important cellular process through which defective/damaged organelles and proteins can be broken down and recycled. The accumulation of these dysfunctional proteins and organelles due to an autophagy deficiency could be leading to the onset of premature senescence in PSAF. As a result, it was suggested that the induction of autophagy was essential for the inhibition of premature senescence. Indeed, the inhibition of autophagy in Ubisol-Q_10_ treated PSAF resulted in the return of the SIPS phenotype, indicating the importance of autophagy induction in Ubisol-Q_10_′s neuroprotective effect. Overall, the various deficiencies associated with the PS-1 mutation in fibroblasts including increased oxidative stress, autophagy inhibition, and premature senescence were overcome following Ubisol-Q_10_ treatment. Another study used NHF and transformed human embryonic kidney (HEK293) cells challenging them with apoptosis-inducing external oxidative stress [86]. Water-soluble CoQ_10_ was able to quench the external ROS allowing the cells to maintain their mitochondrial membrane potential under otherwise lethal doses of H_2_O_2_. The pro-apoptotic activity of Bax was inhibited by Ubisol-Q_10_ treatment through the potent quenching of ROS and the reduction of cytochrome-C release triggered by Bax insertion into the mitochondrial membrane. This study indicates that Ubisol-Q_10_ supplementation can stabilize mitochondria while inhibiting oxidative stress-induced apoptosis in neuronal cells, further supporting its role as a neuroprotective agent.

More recently, the same mechanism of autophagy induction observed in PSAF was observed in PQ exposed rats [87]. Beclin-1 protein levels were elevated in the brains of PQ exposed rats given Ubisol-Q_10_ supplemented water compared to rats given plain drinking water. Furthermore, levels of oxidative stress as indicated by lipid peroxidation product 4-hydroxynonenal (4-HNE) were reduced in PQ exposed rats given Ubisol-Q_10_ further reinforcing the potent antioxidant properties of Ubisol-Q_10_. Microglia activation and astroglia activation was also observed in the brains of PQ exposed rats indicating anti-inflammatory properties of Ubisol-Q_10_.

Results from in vitro studies provided compelling evidence that Ubisol-Q_10_ acted as a potent inhibitor of neuronal cell death caused by excessive oxidative stress, excitotoxicity, or mitochondrial toxins. Its ability to prevent premature senescence in presenilin mutated human fibroblasts and resumption of autophagy is a very significant discovery.

**Figure 2:**
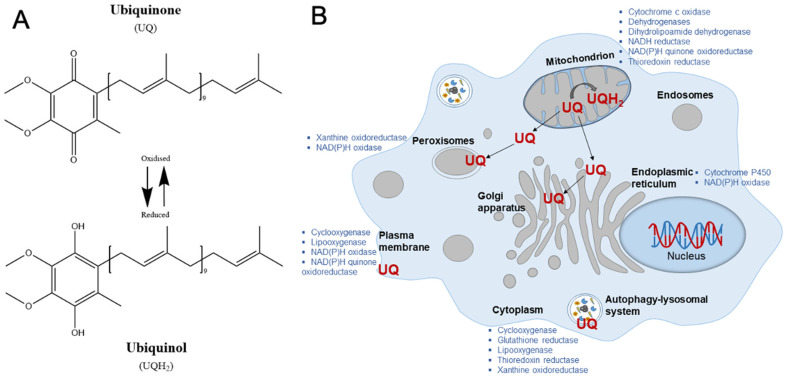
Chemical structure and biological functions of CoQ_10_. (**A**) Enzymatic conversion of coenzyme-Q_10_ from oxidized Ubiquinone (UQ) to reduced Ubiquinol (UQH2) form that is crucial for enzymatic activities of multiple cellular oxidoreductases (depicted in (**B**)). (**B**) Schematic representation of cellular and subcellular organelles indicating enzymatic complexes using CoQ_10_ conversion for their activities (**A**).

The results from the in vitro studies summarized above revealed the involvement of broad-spectrum cellular processes in the observed neuroprotection. CoQ_10_, a lipid soluble electron carrier, can act as either antioxidant or prooxidant during the reversible transition from fully oxidized (ubiquinone) to fully reduced (ubiquinol) form (Figure 2A). Due to its lipophilic nature CoQ_10_ conversion takes place mainly in the milieu of cellular membranes, where it supports the functionality of multiple enzymatic complexes requiring electron transfer reactions; hence, it participates in a multitude of cellular pro-survival metabolic processes (Figure 2B). Clearly, to achieve the optimal responses of these many enzymatic complexes, a sustained supplementation in the range of nano to micromolar is sufficient to achieve this. Therefore, its presence is crucial not only for mitochondrial electron transport chain and cellular energy production, but also for the function of many cellular oxidoreductases [4].

Here, the investigators were faced with a question as to whether Ubisol-Q_10_ with such a broad spectrum of neuroprotective efficacy in vitro could potentially halt the progression of neurodegenerative diseases by protecting neurons in the brain in animal models of PD and AD neurodegenerative diseases. The outcomes of such studies are summarized in the section below.

## 7. Ubisol-Q_10_ Prevents Neuronal Loss and Halts the Progression of Neurodegeneration in Rodent Models of PD

Parkinson’s disease is the second most common neurodegenerative disease characterized by the loss of dopaminergic (DA) neurons in the *substantia nigra pars compacta* (SNpc) region of the brain. Physical symptoms of PD such as bradykinesia, rigidity, postural instability, and resting tremors become obvious when approximately 60–70% of DA neurons in the SNpc are lost [88]. The exact cause of PD development is not known, but a correlation between high exposure to the environmental toxin paraquat and increased risk of PD has been shown [89,90]. Paraquat is known to inhibit complex I of the ETC in mitochondria. Indeed, several chemicals including MPTP (1-methyl-4-phenyl-1,2,3,6-tetrahydropyridine), paraquat, and rotenone that inactivate complex I of the oxidative phosphorylation pathway do induce degeneration of dopaminergic neurons in rodents. It is generally hypothesized that dysfunction of mitochondria, increased ROS burden, and the accumulation of dysfunctional proteins are at the center of PD pathophysiology. Therefore, CoQ_10_ with a critical role in cellular energy production and antioxidant properties could be an excellent therapeutic for PD. Accordingly, studies have shown CoQ_10_ is effective in preventing cell death caused by toxins such as PQ, but only at very high doses of an oil-soluble formulation of CoQ_10_ [91]. This could be due to poor bioavailability in the brain. A water-soluble formulation of CoQ_10_, Ubisol-Q_10_, which is already shown to be extremely effective in protecting neuronal cells in culture against oxidative stress, glutamate excitotoxicity and paraquat toxicity, could be the better solution.

Furthermore, the investigators have demonstrated that Ubisol-Q_10_ was efficiently absorbed into the blood and then into tissues including the brain when administered orally at low doses to mice or rats [62,63]. Feeding of Ubisol-Q_10_ supplemented drinking water to rats prevented Paraquat-induced loss of dopaminergic neurons and motor deficit as a prophylactic and intervention treatment [60]. Furthermore, in an MPTP-induced mouse model of PD, in which MPTP had already initiated neurodegeneration, orally administered Ubisol-Q_10_ blocked the neuronal death pathway allowing the DA neurons to survive as long as the supplementation was continued (for at least 8 weeks post-MPTP treatment) suggesting a potential therapeutic efficacy for PD patients [63]. The neuroprotective Ubisol-Q_10_ treatment triggered an astrocytic activation response indicating the pro-survival role of these cells [63]. The therapeutic neuroprotective effect of Ubisol-Q_10_ was also demonstrated in a Paraquat-induced chronic model of PD in rats [62]. Feeding Ubisol-Q_10_ supplemented water to rats post-Paraquat injection halted the progression of neurodegeneration and ameliorated motor dysfunction [62]. Most importantly, in both models, the effective dose of Ubisol-Q_10_ was only 6 mg/kg/day, far lower than that of the oil-soluble formulation (400–1600 mg/kg/day) [22]. Ubisol-Q_10_ is shown to be safe and has been given GRAS status by the FDA. A genetic susceptibility model of PD in mice was also used and it was found that Ubisol-Q_10_ protects against MPTP-induced neurodegeneration and motor dysfunction in DJ-1 deficient mice [61]. Thus, Ubisol-Q_10_ might be a treatment prospect for people genetically predisposed to PD as well as with sporadic PD. Currently there is no therapeutic available to stop the progressive loss of neurons in PD patients; only symptomatic relief is obtained by levodopa, but prolonged use leads to drug induced dyskinesia, which severely affects the patient’s quality of life [92]. Ubisol-Q_10_ could be a safe nutraceutical therapeutic that has the potential to halt the progression of PD and is a safe material that can be taken over long periods of time.

## 8. Ubisol-Q_10_ Halts Progression of AD Pathology and Symptoms by Inhibition of Oxidative Stress, Inflammation, and Activation of Autophagy

Hippocampal neurodegeneration is a key pathological feature of AD, resulting in severe cognitive impairments [93]. A functional hippocampus is crucial for the formation of long-term memories and the performance of spatial memory tasks [94,95,96]. As a result, any damage to the hippocampal region of the brain, whether it be directly through lesions [97,98,99,100,101] or indirectly through AD development [102,103], drastically influences long-term memory and spatial working memory. This vindicates the usage of behavioral testing of long-term and spatial memory on transgenic rodent AD models to assess potential therapeutic effects. For example, double transgenic mice containing mutations in both the PS-1 and APP genes serve as a useful AD model for examining therapeutic effects in vivo. Despite oil-soluble coenzyme-Q_10_ showing neuroprotection in neurodegenerative rodent models, it failed in clinical trials for Parkinson’s disease, Huntington’s disease, and amyotrophic lateral sclerosis [22,26,104]. Due to its poor bioavailability, its effective dose in rodent models was 200–1600 mg/kg/day, well above FDA-approved limits in humans for clinical trials. Thus, the lower doses administered during clinical trials likely resulted in the lack of neuroprotection observed. However, water-soluble coenzyme-Q_10_, Ubisol-Q_10_, demonstrates very promising results in vivo at doses of just 6 mg/kg/day.

Muthukumaran et al., examined long-term memory in double transgenic AD mice through observing the degree of dishabituation in a Y-maze [58]. Untreated transgenic mice showed a greater spontaneous recovery of a previously habituated exploratory response following a long rest from exposure to the Y-maze compared to wild-type mice. However, oral Ubisol-Q_10_ supplementation in double transgenic mice reduced the spontaneous recovery compared to the untreated group indicating improved memory processes following treatment. They also conducted novel location / novel object recognition (NL/NOR) testing where they found untreated transgenic mice explored a moved familiar object less than Ubisol-Q_10_ treated transgenic and wild-type mice indicating decreased memory of the object’s spatial features due to hippocampal neurodegeneration [58].

Confirmation of neuronal preservation was seen through NeuN immunohistochemical staining which qualitatively demonstrated higher neuron counts in Ubisol-Q_10_ treated transgenic brains compared to untreated mice. Biochemically, Ubisol-Q_10_ reduced the circulating amyloid-beta and amyloid-beta plaque levels in the hippocampus characteristically observed in AD patients [58]. Due to autophagy’s role in clearing damaged/defective proteins and organelles, it was questioned as to whether autophagy activation could be important for the clearing of these amyloid-beta plaques in transgenic AD mice. Indeed, it was shown that autophagy is inhibited in the brains of the double transgenic mice but is activated following Ubisol-Q_10_ treatment [59]. Furthermore, pro-survival astrocytes were activated in the brains of double transgenic mice following Ubisol-Q_10_ supplementation while reactive microglial clumping around amyloid-beta plaques was drastically reduced in a manner similar to the wild-type mice. The NLRP3 (nucleotide-binding and oligomerization domain (NOD)-like receptor family, pyrin domain containing 3) inflammasome pathway is activated in immune and glial cells in response to sterile triggers such as dying neurons, cytokines and chemokines, mitochondrial ROS, lysosomal disruption, extracellular glutamate, K^+^ efflux and Ca^2+^ influx. Upon sensing cellular stress, NLRP3 recruits an adaptor protein (ASC) that activates caspase-1 leading to the maturation and secretion of pro-inflammatory cytokines, IL-1β and IL-18 [105]. Oral administration of 300 mg/day CoQ_10_ (soft gel capsules, Pharma Nord) for 40 days in patients suffering from fibromyalgia (*n* = 20) reduced serum levels of IL-1β and IL-18 [106]. In addition, Peng et. al., have recently shown that intraperitoneal injections of idebenone (an artificial derivative of CoQ_10_) at 100 mg/kg in an animal model of ischemic-reperfusion injury attenuated cerebral inflammatory responses by dampening microglial NLRP3 inflammasome activity [107]. Overall, Ubisol-Q_10_ treatment was successful in reversing many AD hallmarks including spatial memory impairments, amyloid-beta plaque formation, neuronal loss in the hippocampus, pro-neuroinflammatory microglial activation, neuroprotective astroglia inhibition, and autophagy deficiencies. As current treatments such as cholinesterase inhibitors and N-methyl-D-Aspartate (NMDA) antagonists strictly provide symptomatic relief, Ubisol-Q_10_ given as a simple oral supplement could serve as a potential therapeutic to halt the progression of AD.

## 9. Astrocytic Responses in the Brains of Ubisol-Q_10_ Treated Animals

**Figure 3:**
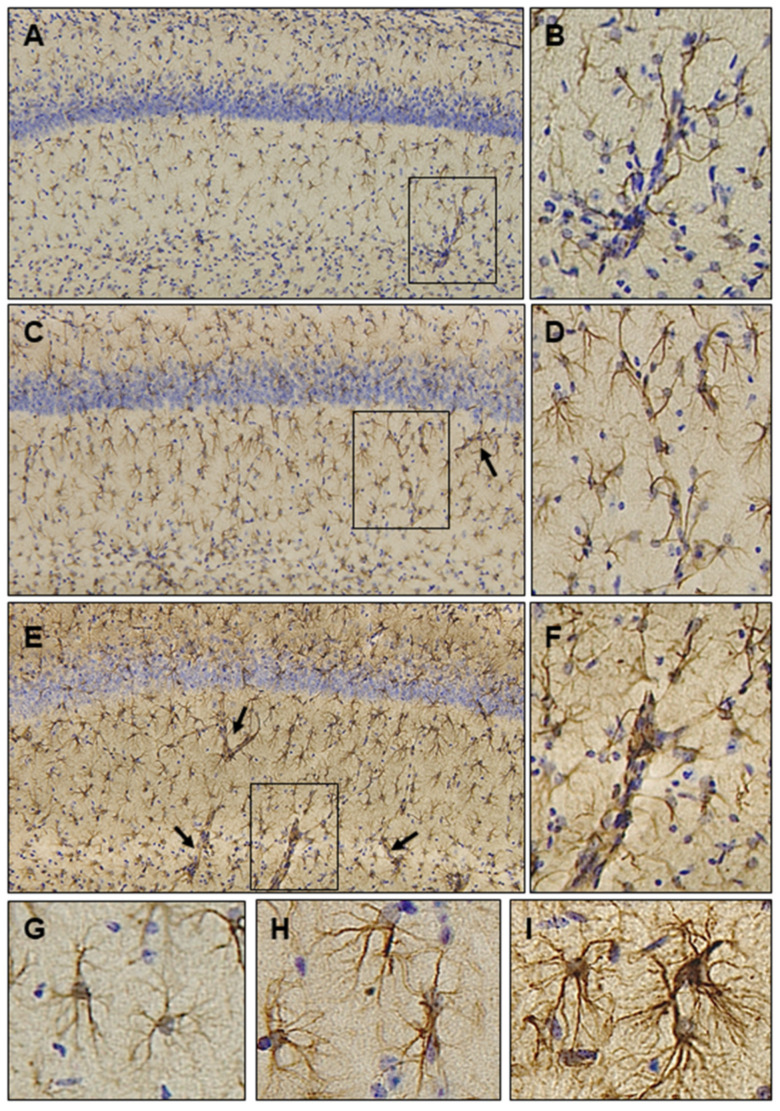
Astrocytic responses to Ubisol-Q_10_ supplementation in the MPTP model of Parkinson’s disease. Formalin fixed free-floating sections were subjected to immunohistochemistry using an antibody raised against glial fibrillary acidic protein (GFAP), a widely used marker of astrocytes. Astrocytes were identified by a brown precipitate at the site of antigen-antibody reaction. Nuclei were counterstained blue with hematoxylin. Shown are photomicrographs at the level of hippocampus from control (**A**), MPTP-injected (**C**) and MPTP-injected mice receiving Ubisol-Q_10_ (**E**); Magnification = 20×. Arrows in panels A, C and E depict cerebral microvasculature with increased GFAP staining forming the perivascular astrocyte endfeet. Boxed areas are represented at a higher magnification—control (**B**), MPTP-injected (**D**) and MPTP-injected mice receiving Ubisol-Q_10_ (**F**). High magnification image of activated astrocyte morphology with increased GFAP staining is shown in MPTP-injected (**H**) and MPTP-injected mice receiving Ubisol-Q_10_ (**I**) as compared to controls (**G**).

As elucidated above, preclinical evidence for the extraordinary efficacy of Ubisol-Q_10_ in protecting neurons and halting the ongoing neurodegenerative processes in the cellular and rodent models of AD and PD has been provided. Astrocytes are the most abundant type of glial cells in the brain that play a pivotal role in regulating almost all homeostatic functions, including maintenance and regulation of the extracellular microenvironment, blood-brain barrier integrity, cerebral blood flow, antioxidant and trophic factor support, uptake and recycling of neurotransmitters as well as detoxification of ROS [108,109,110]. Accordingly, histopathological studies on the brain tissues were carried out and astrocytic responses to Ubisol-Q_10_ supplementation was examined. Microscopic examination revealed a robust increase in astrocytic GFAP expression in the brains of MPTP-injected animals receiving Ubisol-Q_10_ (Figure 3). Interestingly, astrocyte activation was seen in all the examined brain regions, including hippocampus, cortex, substantia nigra and striatum, not limited only to the diseased brain region. Similar changes in astrocytic cells were also seen in rats receiving Ubisol-Q_10_ and challenged with paraquat.

Under higher magnification, astrocytic processes appeared longer and extensively ramified, extending GFAP-positive hypertrophic processes and contacting neighboring astrocytes (Figure 3E,I), possibly establishing an astrocytic syncytial network. Many studies have highlighted the significance of this highly specialized intercellular machinery in facilitating communication through proteins called gap junctions [111]. These communication channels allow the direct passage of ions (Ca^2+^, Na^+^ and K^+^), small molecules (glucose, water, metabolites, second messengers and neurotransmitters) and growth factors to support neuronal function [112]. The astrocyte syncytium is also thought to be involved in the detoxification of excess extracellular glutamate, protecting neurons from excitotoxic damage [113]. Furthermore, astrocytes engage in an intensive bidirectional crosstalk with neurons to regulate synaptic activity. Recently, a plethora of evidence has emerged supporting the role of astrocytes as significant modulators of neuronal circuits, networks, and synaptic neurotransmission. Indeed, astrocytes wrap around neuronal synapses forming the ‘tripartite synapse’ comprising of pre- and post-synaptic neurons and the astrocyte [114]. Astrocytes use intracellular Ca^2+^ transients as their encoding language to communicate complex signals to neurons in a highly synchronized bursting behavior via neuronal networks and regulate synaptic transmission. Using co-cultures of astrocytes and neurons that recapitulates neuronal network synchronization and connectivity, it has been shown that astrocytes are essential for the development of synchronized activity [115]. Furthermore, the spike and burst rates were reduced in matured networks of glutamatergic neuron-astrocyte co-cultures as compared to monolayer cultures [116]. Similarly, numerous studies using human induced pluripotent stem cells (iPSC)-derived neurons have demonstrated an increased maturation of network functionality and synchronization in the presence of astrocytes [117,118]. Together, these results demonstrate that astrocytes play an important role in neuronal firing synchronicity and synaptic coordination. It has been hypothesized that selective loss of neurons during disease progression results in disintegration of neuronal networks and abnormal synchronization. Supplementation with Ubisol-Q_10_ could help in neuronal firing synchronicity and synaptic coordination leading to stabilization of neural activities.

Astrocytes are also known to establish a bidirectional communication with the Blood-Brain-Barrier (BBB) which plays an important role in the maintenance of CNS homeostasis and neuronal activity [119]. An increase in GFAP-expressing astrocytic endfeet was seen extending into the cerebral blood vessels in animals receiving Ubisol-Q_10_ as compared to toxin-treated or control animals (Figure 3B,D,F). It is plausible that an increased neurovascular and neurometabolic coupling [120] was established between astrocytes and brain endothelial cells resulting in increased cerebral blood flow and preservation of BBB function [110]. It is noteworthy that astrocytes rely on mitochondrial oxidative phosphorylation for ATP production and Ubisol-Q_10_ potentially enhanced their ability to synthesize ATP and maintain neuronal bioenergetics. Indeed, astrocytes act as gatekeepers and protect neurons from oxidative and nitrosative stress [121]. Taken together, studies support the notion that astrocytes could be active players in the neuroprotection observed in these experimental models.

Mounting evidence indicates that activated astrocytes may initiate pathological processes by secreting neurotoxic factors such as ROS and pro-inflammatory cytokines (IL-1β, IL-6 and TNF-α) that likely contribute to neuroinflammation and neurodegeneration [122]. Exposure of mixed neuron-astrocyte cultures derived from human NT2/D1 cells to toxic levels of glutamate resulted in oxidative stress and neurotoxicity which was suppressed by pre-treatment with Ubisol-Q_10_ [83]. In addition, cortical neurons co-cultured with senescent astrocytes showed increased susceptibility to glutamate toxicity, possibly due to decreased efficiency to detoxify glutamate [123]. Oil-soluble CoQ_10_ was also shown to protect cultured murine astrocytes from ROS-induced (Ultraviolet-B radiation) damage by targeting mitochondrial function and oxidative stress [124]. An increase in markers of spinal oxidative stress, neuroinflammation (astrocyte and microglial activation) and hyperalgesia was seen in sickle mice, which were remarkably decreased with administration (by gavage) of oil-soluble CoQ_10_ at 45 mg/kg daily for 4 weeks [125]. Thus, maintenance of active astrocyte-neuron communication is important for neuronal survival and function. CoQ_10_ functions as a scavenger of ROS and an important antioxidant by activating antioxidant enzymes through Nrf2 signaling and protecting neuronal cells from oxidative stress.

Increasing evidence suggests that CoQ_10_ can also function as an anti-inflammatory molecule by modulating nuclear factor kappaB (NFĸB)-dependent gene expression. Consequently, treatment of human and murine macrophage and monocytic cell lines with lipopolysaccharide induced secretion of various cytokines and chemokines which were significantly reduced in the presence of CoQ_10_ [126,127]. Similarly, exposure of rat pheochromocytoma PC-12 cells to β-amyloid (25–35) induced cell death and increased the production of pro-inflammatory lipid mediators, cyclooxygenase-2 (COX-2) and prostaglandin E2 (PGE2), respectively. Pre-incubation of these cultures with CoQ_10_ decreased COX-2 and PGE2 levels, mainly by blocking nuclear translocation of NFĸB and inhibiting pro-inflammatory gene expression [128]. CoQ_10_ can act as an antioxidant and an anti-inflammatory molecule to protect astrocytes from oxidative damage and neuroinflammation therefore enhancing their ability to protect neurons, in turn, leading to amelioration of pathological symptoms.

## 10. Ubisol-Q_10_ Effectiveness in Contrast to Other Coenzyme-Q_10_ Formulations

**Figure 4:**
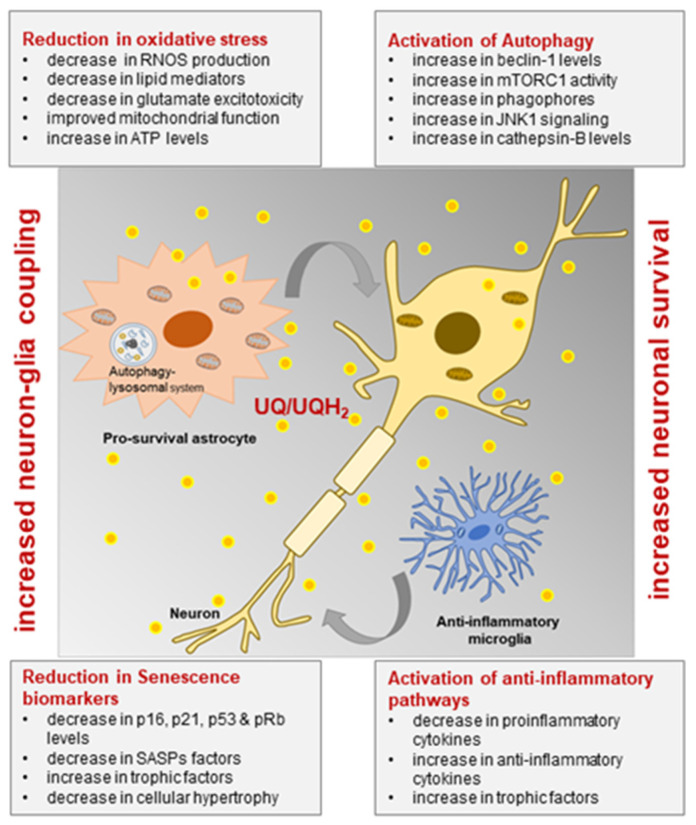
Schematic illustrating the neuroprotective potential of Ubisol-Q_10_. Ubiquinone (UQ) is converted to Ubiquinol (UQH2), which is involved in key cellular functions. Neuroprotection could be mediated through modulation of several astrocytic and microglial pathways, for instance reduction of oxidative stress by decreasing reactive oxygen and nitrogen species (RNOS), lipid mediators, glutamate excitotoxicity and improving mitochondrial function. Activation of autophagy by increasing beclin-1 levels and up-regulating mTORC1, JNK and cathepsin-B activity. Decreasing cellular senescence by reducing growth arrest and secretion of pro-inflammatory mediators and increasing secretion of neurotrophic growth factors. Increasing anti-inflammatory effects by decreasing inflammatory mediators such as IL-6, TNF-α and IL-1β. These effects result in restoring cellular homeostasis, leading to increased neuron-glia coupling and neuronal survival.

The reasons for failed clinical trials with oral supplementation of oil-soluble CoQ_10_ formulations are unclear, particularly since the bioactive component of tested formulations is coenzyme-Q_10_, the same molecule used in Ubisol-Q_10_. However, Ubisol-Q_10_ also contains PTS, a prodrug form of vitamin E. It should be noted that no clinical trials have reported the therapeutic efficacy of α-tocopherol alone [129]. Taking all published data into consideration, one explanation that comes to mind is the difference in quantities of supplied CoQ_10_. In some of the clinical trials mentioned above, extremely large doses of CoQ_10_ were used, and yet no benefits were seen from such applications. Interestingly, micromolar concentrations of Ubisol-Q_10_ have shown remarkable neuroprotection both in vitro and in vivo, possibly by improving cellular metabolism (Figure 4) (Table 1) [57,58,59,60,61,62,63]. In each of these in vivo studies, drinking water was supplemented with 50 µg/mL CoQ_10_ and 100 µmol/mL of PTS (of which 30% is converted to vitamin E) or the equivalent of 6 mg/kg body weight. This was sufficient to penetrate the brain as demonstrated by a remarkable neuroprotection [57,58,59,60,61,62,63] and the engagement of astroglia within the brain parenchyma (Figure 3). To achieve such effects, this supplementation must be sufficient to mobilize cellular metabolism and cyto-protection. As depicted in Figure 4, these metabolic outcomes must be a result of multiple cellular enzymatic complexes that use the conversion of ubiquinone to ubiquinol and are localized to various specialized subcellular compartments. Clearly, micromolar concentrations of Ubisol-Q_10_ are sufficient to achieve this high efficacy.

The neuroprotective ability of Ubisol-Q_10_ has been demonstrated through both in vitro and in vivo studies [57,58,59,60,61,62,63]. All the data indicates just how effective it could be as a therapeutic treatment if biologically relevant doses were available for patients. A presented model based on these results and the literature on CoQ_10_ is depicting potential mechanisms of the neuroprotective functions of Ubisol-Q_10_ (Figure 4).

## 11. Conclusions and Future Prospects

It is now well established that CoQ_10_ has a great therapeutic potential for neurodegenerative diseases. However, due to poor bioavailability, an approach was taken to use very high doses of oil-soluble CoQ_10_ (400–1600 mg/kg/day which translates to 28–114 g/day for a 70 kg patient) in rodent models of neurodegenerative diseases. Clinical trial results did not show any efficacy as a maximum of only 2.4 g/day was used in the study [22,26]. On the other hand, water-soluble Ubisol-Q_10_ with an effective dose of 6 mg/kg/day (translating to 420 mg/day for a 70 kg patient, well within the recommended range of CoQ_10_ dosing by the FDA), which was efficient in halting the progression of neurodegeneration in both rodent models of PD and AD, would be an optimal candidate for clinical studies. It is important to note that oral administration of Ubisol-Q_10_, a GRAS approved material led to reduced oxidative stress, activation of autophagy, activation of pro-survival astroglia, and inhibition of cell death in addition to the amelioration of behavioral deficits in rodent models of AD and PD. Neurodegenerative diseases such as AD and PD are progressive debilitating diseases spanning over long periods of time. Any therapeutic intervention must be non-toxic and without any side-effects as a long-term treatment. Ubisol-Q_10_, a safe nutraceutical material with great preventative and therapeutic potential could be the ideal candidate for development as an AD and PD treatment.

Ubisol-Q_10_ can be easily produced; although it requires commercial manufacturing of PTS, its synthesis is not complicated, and formulation production is very simple (refs patent and Sikorska et al.). Both compounds of Ubisol-Q_10_ are readily absorbed in the body and find their way into the brain within 1 h of oral delivery where they participate in evoking powerful neuroprotection. They do not accumulate in tissues but are present for as long as they are continuously supplied. This seems to be a perfect solution for the treatment of ongoing neurodegeneration, and it will help millions of individuals for whom there is currently no hope. It should be clearly highlighted that the lack of beneficial results from the studies using a huge amount of CoQ_10_ suggest that an incorrect approach was taken in clinical trials and the studies should be repeated using less and not more of this bioactive compound. It has been demonstrated that use of micromolar concentrations of Ubisol-Q_10_ seems to be sufficient to successfully interfere with neurodegeneration. As we understand that these claims need to be further validated through more preclinical and clinical studies, and to our knowledge there is no other CoQ_10_ formulation suitable for parenteral delivery of CoQ_10_, it is our hope to find enough open-mindedness in the medical and pharmaceutical industries willing to test this further, particularly since currently there are no effective treatments to halt the progression of neurodegenerative diseases.

## Figures and Tables

**Table 1:** Summary of recent research progress made with various formulations of coenzyme-Q_10._

	Neurodegenerative Disease	Model	Effective Dose	Mode of Administration	Major Outcomes	Reference
**Oil-Soluble CoQ_10_**	Alzheimer’s Disease	In-Vivo (Mice)	10 g/kg diet	Oral	- Protection against neurotoxicity & oxidative stress - Mitochondrial stabilization - Reduced Aβ plaques - Improved cognitive performance	Wadsworth 2008 [30]
		In-Vitro	6.25 µM	Media Supplementation		Wadsworth 2008 [30]
		In-Vivo (Mice)	0.4% or 2.4%	Oral		Dumont 2011 [35]
		In-Vitro	10 µM	Media Supplementation		Sadli 2013 [33]
	Amyotrophic Lateral Sclerosis (ALS)	In-Vivo (Rats & Mice)	200 mg/kg/ day	Oral	- Anti-oxidative effects - Preserved mitochondrial function - Increased lifespan	Matthews 1998 [39]
		In-Vivo (Mice)	200 mg/kg/day (no effect)	Oral (Gavage)		Lucchetti 2013 [44]
	Frontotemporal Dementia	In-Vivo (Mice)	0.5% of Diet	Oral	- Improved behaviour & survival	Elipenahli 2012 [50]
	Huntington’s Disease	In-Vivo (Rats)	200 mg/kg/day	Oral	- Improved motor performance & survival - Delayed weight loss - Prevented striatal neuron intranuclear inclusion formation - Slowed striatal neuron atrophy - Reduced HTT aggregate formation - Reduced oxidative damage	Matthews 1998 [39]
		In-Vivo (Mice)	400 mg/kg/day	Oral		Ferrante 2002 [40]
		In-Vivo (Mice)	0.2% of Diet	Oral		Stack 2006 [41]
		In-Vivo (Mice, Rats)	1600–2000 mg/kg/day	Oral		Yang 2009 [24]
		In-Vivo (Mice)	0.2% of Diet	Oral		Hickey 2012 [43]
	Machado-Joseph Disease	In-Vitro	10 µM, 30 µM, 90 µM	Media Supplementation	- Improved cell viability & reduced apoptosis - Prevented ATX3 protein aggregation	Lopes-Ramos 2016 [51]
	Multiple-System Atrophy	In-Vitro	25 µM	Media Supplementation	- Improved oxidative metabolism - Reduced apoptosis	Nakamoto 2018 [53]
	Parkinson’s Disease	In-Vivo (Mice)	200 mg/kg/day	Oral	- Dopaminergic neurons saved in striatum and SNpc - Clearance of α-synuclein aggregates - Limited oxidative damage - Reduced pro-inflammatory cytokines	Beal 1998 [21]
		In-Vivo (Mice)	200–1600 mg/kg/day	Oral		Cleren 2008 [22]
		In-Vivo (Mice)	1% of Diet	Oral		Yang 2009 [24]
		In-Vivo (Drosophila)	100 mg/mL (no effect)	Oral		Faust 2009 [25]
		In-Vivo (Rats)	25 µg/mL	Intrastriatal Injection		Park 2020 [27]
**Ubisol-Q_10_**	Alzheimer’s Disease	In-Vitro	50 µg/mL	Media Supplementation	- Inhibited oxidative stress - Upregulated autophagy - Maintained MMP - Reduced cell cycle arrest protein expression - Prevented SIPS onset - Inhibited apoptosis - Improved memory - Reduced hippocampal neurodegeneration - Cleared Aβ plaques - Increased astrocyte activity - Reduced microglial activity	Ma 2014 [57]
		In-Vivo (Mice)	6 mg/kg/day	Oral		Muthukumaran 2018 [58]
		In-Vivo (Mice)	50 µg/mL	Oral		Vegh 2019 [59]
		In-Vitro	50 µg/mL	Media Supplementation		Vegh 2019 [59]
	Parkinson’s Disease	In-Vivo (Rats)	50 µg/mL	Oral	- Reduced oxidative stress - Maintained ATP generation - Stabilized mitochondrial membrane - Prevented loss of dopaminergic neurons - Activated pro -survival astrocytes - Ameliorated motor dysfunction	Somayajulu-Nitu 2009 [60]
		In-Vivo (Mice)	6 mg/kg/day	Oral		Muthukumaran 2014 [61]
		In-Vivo (Rats)	6 mg/kg/day	Oral		Muthukumaran 2014 [62]
		In-Vivo (Mice)	3 mg/kg/day	Oral		Sikorska 2014 [63]
